# Medicinal Leeches to Aid in Post-procedural Hematoma Evacuation

**DOI:** 10.7759/cureus.43338

**Published:** 2023-08-11

**Authors:** Karolina Y Marquez-Gil, Elvis Mesa, Nabeel Kouka, Ilya Fonarov, Damian Casadesus

**Affiliations:** 1 Medicine, St. George's University School of Medicine, New York, USA; 2 Medicine, Jackson Memorial Hospital, Miami, USA; 3 Neuromusculoskeletal Medicine, Salus Neuromuscular Institute, Hallandale Beach, USA; 4 Hospital Medicine, Jackson Memorial Hospital, Miami, USA; 5 Internal Medicine, Jackson Memorial Hospital, Miami, USA

**Keywords:** hirudotherapy, leech saliva, rapid wound healing, delayed wound healing, hormonal therapy, infection complications, anticoagulant properties, medicinal leech therapy, hematoma formation, von willebrand disease

## Abstract

Patients presenting with a post-invasive procedure hematoma can be treated with medicinal leeches to evacuate the hematoma. Our patient, a postmenopausal woman in her 60s, with a past medical history of hypothyroidism, presented to the outpatient clinic with pain, redness, warmth, and swelling on her right thigh. Ten days prior, the patient had undergone a subcutaneous pellet implant procedure in the right thigh for hormonal replacement therapy. The patient developed post-procedure cellulitis and soft tissue infection and was treated with antibiotics. The patient developed a progressively enlarged hematoma at the implant site. The hematoma was treated with medicinal leeches. Two weeks after treatment, the implant area healed. The patient had a family history of von Willebrand disease and a history of prolonged bleeding during childbirth, menstruation, and dental procedures. A von Willebrand panel was obtained, and the results were consistent with a new diagnosis of von Willebrand disease.

## Introduction

Medicinal leech therapy (MLT), known as hirudotherapy (HT), is a highly effective method for alleviating venous congestion. The principles behind the effectiveness are found in the leeches' capacity to drain blood and reduce hematomas and venous congestion. Biomolecular analyses of leeches' saliva have shown multiple compounds with antithrombotic, anticoagulant, anti-inflammatory, antimicrobial, and pain-relieving properties [1].

The underlying mechanism behind leech therapy can be attributed to two main factors. First, leech saliva contains a potent anticoagulant called hirudin, which prevents blood from clotting, promotes blood flow, and prevents the formation of additional blockages. Second, leeches naturally extract blood through suction, effectively reducing the pressure in the affected area [2]. With the combination of these mechanisms, leech therapy offers a complete approach to addressing venous congestion and its complications.

## Case presentation

A postmenopausal woman in her 60s with a past medical history of hypothyroidism on levothyroxine presented to the outpatient clinic with pain, redness, warmth, and swelling on her right thigh. Ten days prior, the patient had undergone a subcutaneous pellet implant procedure in the right thigh for hormonal replacement therapy (total of five pellets implanted: three testosterone, one progesterone, and one estrogen) for postmenopausal symptoms of skin dryness, loss of hair, hot flashes, insomnia, and difficulty concentrating. The patient had a family history of von Willebrand disease. Nonetheless, no formal testing has been conducted on the patient. However, they did report prolonged bleeding during childbirth, menstruation, and dental procedures.

Upon the physical examination, the patient was afebrile with normal vital signs. The implant site on the right lateral thigh was edematous, erythematous, and tender to palpation, with an opening draining purulent drainage. The rest of the physical exam was unremarkable.

Following the placement of the hormonal pellets and subsequent infection, laboratory findings were observed in Table 1. These laboratory findings provide essential baseline information for the patient's health status pre- and post-procedure (four weeks) and help guide subsequent medical interventions.

**Table 1:** Relevant laboratory values before and after hormone pellets with reference TSH: Thyroid Stimulating Hormone, DHEA: Dehydroepiandrosterone

Test	Patients’ value (Prior to procedure)		Test	Patients’ value (4 weeks following the procedure)		Normal value
White blood count	4.7 x10³/mcL		White blood count	4.4 x10³/mcL		3.8-10.8 x10³/mcL
Hemoglobin	10.7g/dL		Hemoglobin	10.6 g/dL		11.7-15.5 g/dL
Hematocrit	33.8%		Hematocrit	33.6%		35.0-45.0%
Platelets	320 x10³/mcL		Platelets	288 x10³/mcL		140-400 x10³/mcL
TSH	3.4 mIU/L		TSH	3.2		0.40-4.50 mIU/L
Sex hormone-binding globulin	105 nmol/L		Sex hormone-binding globulin	90		14-73 nmol/L
Follicle-stimulating hormone	85.0 mIU/mL		Follicle-stimulating hormone	38.3		23.0-116.3 (Postmenopausal)
DHEA sulfate	59 mcg/dL		DHEA sulfate	55		9-118 mcg/dL
Estradiol	<15 pg/mL		Estradiol	46		< or = 31 pg/mL (Postmenopausal)
Total testosterone	11 ng/dL		Total testosterone	251		2-45 ng/dL
Free testosterone	0.6 pg/mL		Free testosterone	20.6		0.1-6.4 pg/mL
Total 25-hydroxy vitamin D	40 ng/mL		Total 25-hydroxy vitamin D	50		30-100 ng/mL

**Figure 1:**
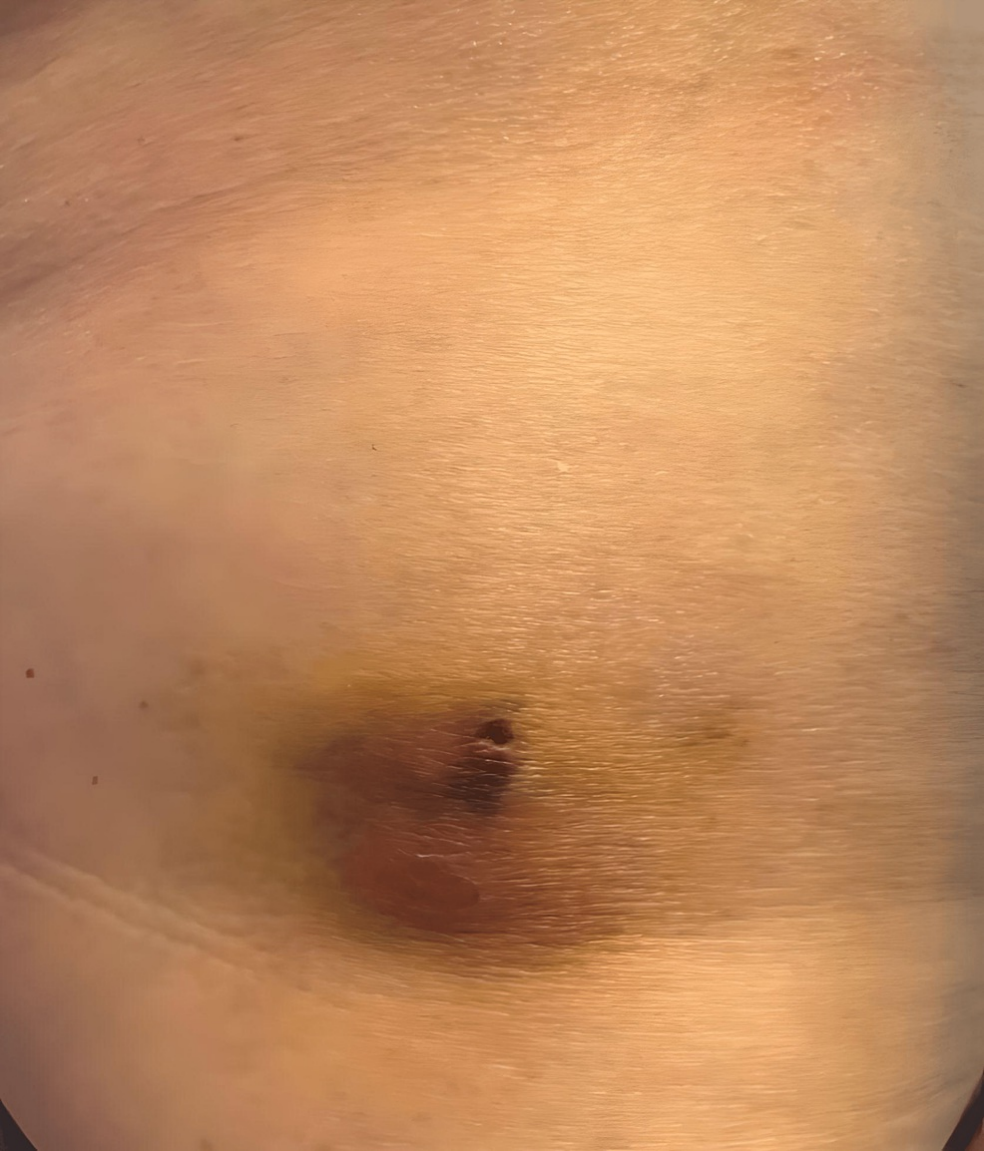
Hematoma on the right lateral thigh

**Figure 2:**
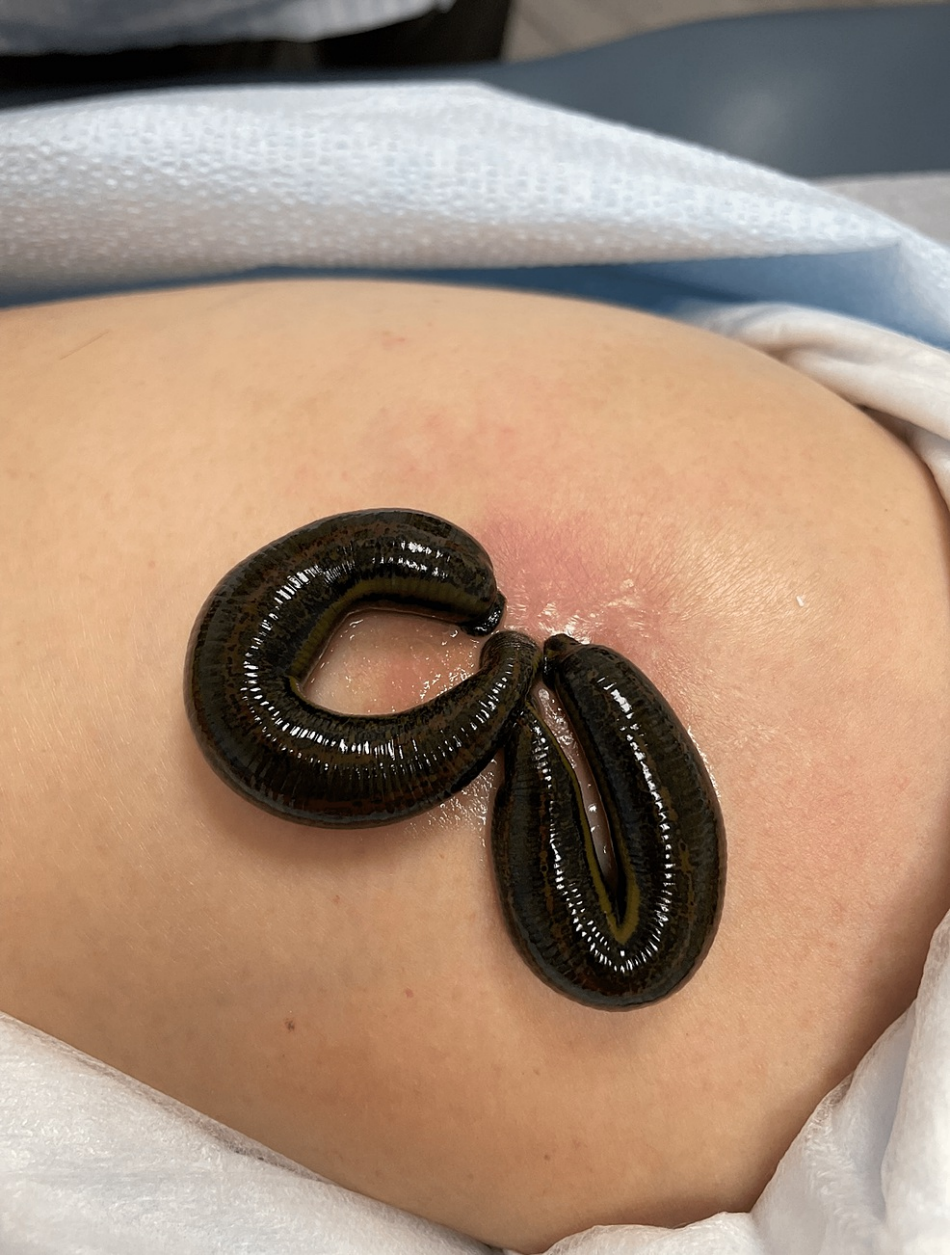
Medical leeches used in the treatment of the hematoma

**Figure 3:**
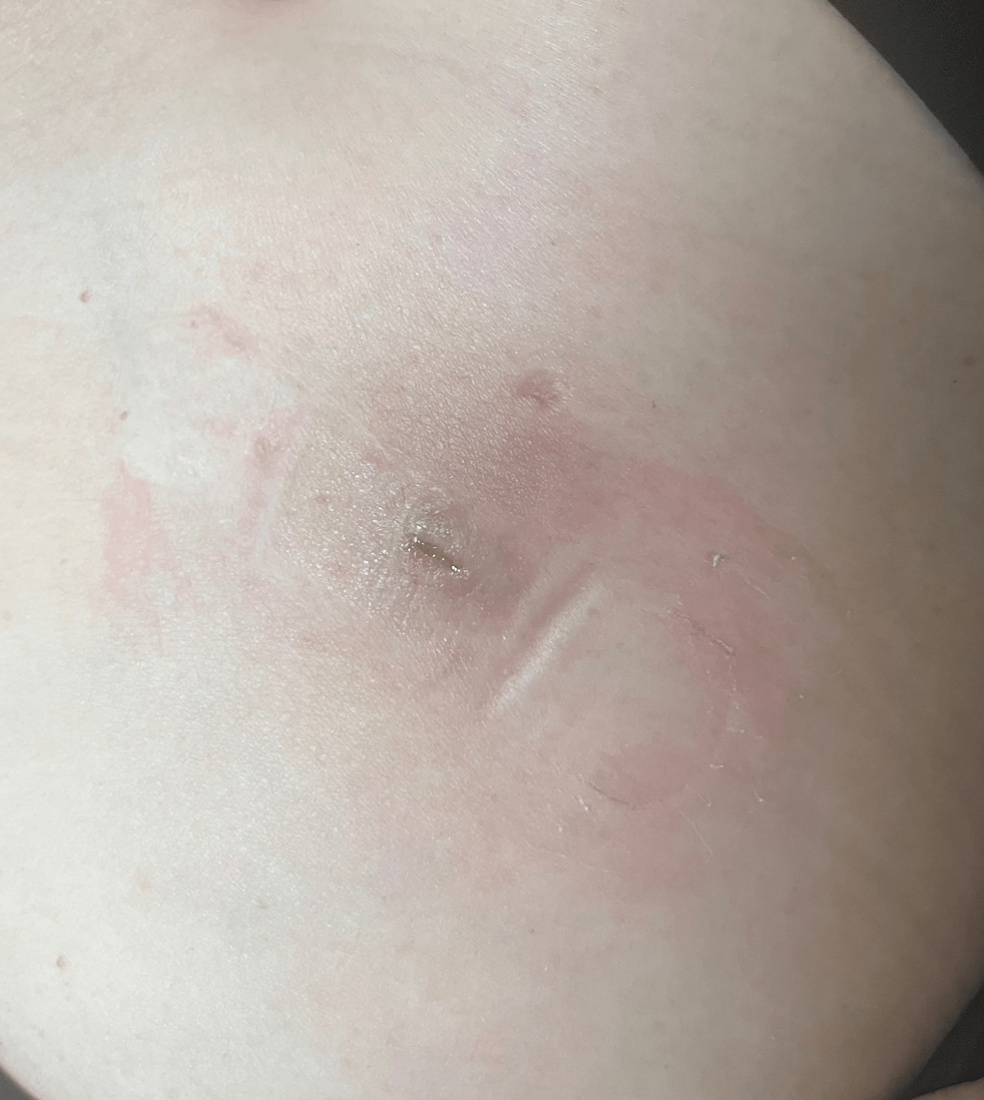
Resolution of the hematoma

After hormone pellet insertion, the patient experienced an infection requiring, antibiotics. Specifically, the patient was prescribed oral ciprofloxacin at a dose of 750 mg twice daily for eight days and received a single intramuscular injection of ceftriaxone 250 mg. Over the next 10 days, a hematoma at the insertion site progressively enlarged (Figure 1). To drain the sanguineous fluid from the hematoma, two medicinal leeches were applied for 45 minutes (Figure 2 and Video 1). Following the treatment, there was a significant improvement in erythema, warmth, swelling, and pain. Complete wound healing was achieved within two weeks after the treatment (Figure 3). Relevant laboratories were monitored after the procedure (Table 1).

**Video 1:** Medical leeches used in the treatment of the hematoma

## Discussion

The mechanisms of medicinal leeches are related to the healing properties of the bioactive compounds in leech saliva. Although more than 100 bioactive substances have been observed in leech saliva, some with a crucial role have been recalled [2,3]. Hirudin, destabilize, and gelin have anticoagulant properties. Bdellins, hirustatin, and eglins have analgesic and anti-inflammatory effects. Hyaluronidase and collagenase are vital to the phases of tissue remodeling. The presence of vasodilators, such as acetylcholine and histamine-like substances, improves blood flow, assisting the healing process [2,3]. Ultimately, natural antimicrobials (chloromycetin and destabilize) with bacteriostatic properties might help reduce infections [2].

Our patient experienced a significant decrease in hematoma size, which can be attributed to the anticoagulant substances in leech saliva. This helps relieve pressure in the capillaries, promoting lymph flow and thus reducing venous congestion. Similarly, vasodilators such as acetylcholine and histamine, help deliver oxygen, growth factors, and nutrients, enabling wound healing [4]. Various studies show MLT with excellent outcomes. Reconstructive and plastic surgery reports have shown 65%-80% salvage rates in congested skin flaps, avulsed digits, ears, and noses, with a risk of loss due to post-operative venous congestion [4].

Riede et al. documented using medicinal leeches in reconstructive surgery for 23 patients with necrotic skin flaps and hematomas. On average, 2.6 leeches were applied to patients' wounds, with a mean of 1.7 treatment sessions per patient. The author reported that 87% of these patients showed restitution and integration of their skin flaps after leech therapy. Clinical improvement was seen after 1.1 days on average [5]. Although prolonged bleeding lasted up to two days, no further complications were observed.

Data in the literature suggest that bleeding and infection are the most common complications of hirudotherapy [6]. The event of infection after the leech application occurs at a 2-36% rate. Aeromonas hydrophila infections are a complication of postoperative leech application. Reports have shown that using leeches without antibiotic treatment can cause bacterial infection at a rate of 20% [6]. Our study corroborates the importance of choosing the appropriate antibiotic prophylaxis due to drug resistance. Our patient received dual therapy (ceftriaxone and ciprofloxacin) to avoid this treatment-related infection.

After leech therapy, there is a potential concern for excess bleeding. Our patient's history of prolonged bleeding during childbirth, menstruation, and dental procedures indicated a higher risk of experiencing excessive bleeding. Therefore, precautions were taken by monitoring pre- and post-procedure hemoglobin. Reviewing the literature, we found a case where the bite wound was treated with QuikClot gauze, which allowed for rapid hemostasis without rebleeding [7]. Therefore, a hemostatic dressing could be a prophylactic measure we could have used in our patient due to her potential risk of prolonged bleeding.

Leech therapy has several benefits; however, there are contraindications in certain groups such as children, pregnant or lactating women, and immunocompromised patients. Besides, it should be avoided in hematological conditions such as hemophilia, severe anemia, active bleeding, and anticoagulant therapy [6].

Our patient was postmenopausal and chose hormonal therapy via implant pellets. The patient had five pellets implanted; three testosterone, one progesterone, and one estrogen. Testosterone replacement therapy benefits musculoskeletal health, cognitive function, and mood in perimenopausal and menopausal women [8]. Our patient was followed up four weeks after the procedure to monitor serum hormone levels (Table 1). Total testosterone was considerably elevated (251 ng/dL). This increase in testosterone was expected. According to a retrospective review, the target testosterone level for women after pellet insertion was 150-250 ng/dl [9]. This study examined more than 1 million hormonal implant procedures and found that the incidence of cellulitis and infection in females was less than 0.1% [9].

Our patient developed a hematoma and infection following pellet insertion, conventional antibiotic treatment did not lead to resolution. However, there was a significant improvement upon introducing medicinal leech therapy, relieving the hematoma, and enabling the healing process. Furthermore, after alleviating the hematoma, prompted by the patient's familial history of von Willebrand disease and the recurrent instances of prolonged bleeding, we conducted a von Willebrand disease (vWD) panel to determine the presence of the disease, potentially contributing to her susceptibility to hematomas. The results, depicted in Table 2, confirmed the presence of vWD. As a next step, it is recommended that the patient consult with hematology specialists to establish an appropriate treatment protocol for managing her vWD condition.

**Table 2:** Von Willebrand panel

Test Name	Patient Range	Reference Range
Factor VIII Activity, Clotting	33	50-180%
Von Willebrand Factor Antigen	44	50-217%
Ristocetin Cofactor	15	42-200%
Partial Thromboplastin Time, Activated	33	23-32 sec

## Conclusions

Medicinal leech therapy can be utilized in patients predisposed to bleeding and delayed wound healing. In this case, the site of the hormone pellet implant was complicated by infection and hematoma. Leech therapy was performed safely and successfully, resulting in complete resolution of the infection, reduction of the hematoma, and rapid wound healing. For patients with platelet disorders predisposing them to developing hematomas, further investigation is needed to determine if medicinal leech therapy can effectively treat this specific patient population. Furthermore, additional studies are encouraged to explore more health benefits of the salivary compounds of leeches.

While the findings suggest the potential of medicinal leech therapy in managing certain post-surgical complications, it is essential to acknowledge the limitations of this study, as it only reported one case. Further research is needed to validate its effectiveness in a broader patient population, especially those with platelet disorders. This case emphasizes the significance of considering unconventional and natural treatments like medicinal leech therapy, particularly when conventional approaches fall short in addressing common post-surgical complications. Exploring alternative options may lead to better patient outcomes and enhanced medical care.
